# Use of a Mobile Biofeedback App to Provide Health Coaching for Stress Self-management: Pilot Quasi-Experiment

**DOI:** 10.2196/41018

**Published:** 2023-04-12

**Authors:** Changwon Son, Sudeep Hegde, Carl Markert, Karim Zahed, Farzan Sasangohar

**Affiliations:** 1 Department of Industrial and Systems Engineering Texas A&M University College Station, TX United States; 2 Center for Critical Care Houston Methodist Hospital Houston, TX United States

**Keywords:** mental health, health coaching, stress, biofeedback, mHealth, mobile apps, breathing exercises, students, veterans, COVID-19, vulnerable population, college student, self-management, mobile health app, psychological well-being, digital health intervention

## Abstract

**Background:**

Mental health is an increasing concern among vulnerable populations, including college students and veterans.

**Objective:**

The purpose of this study was to determine if mobile health technology combined with health coaching can better enable a user to self-manage their mental health.

**Methods:**

This study evaluated the mobile app “Biofeedback” that provided health coaching on stress self-management for college student veterans’ mental health concerns. Twenty-four college student veterans were recruited from a large public university in Texas during the spring 2020 semester, impacted by COVID-19. Ten participants were assigned to the intervention group where they used the mobile Biofeedback app on their smartphones and smartwatches, and 14 were assigned to the control group without the app; assignment was based on mobile phone compatibility. Both groups participated in one initial lab session where they learned a deep-breathing exercise technique. The intervention group was then asked to use the mobile Biofeedback app during their daily lives and a smartwatch, and the control group was asked to perform the breathing exercises on their own. Both groups filled out Patient Health Questionnaire (PHQ-9) and Generalized Anxiety Disorder (GAD-7) self-assessments at 2-week intervals. At the end of the semester, both groups were given an exit interview to provide user experience and perceived benefits of health coaching via the mobile biofeedback app.

**Results:**

The deep-breathing exercise in the initial lab session reduced stress in both groups. Over the course of the study, the app recorded 565 coached breathing exercises with a significant decrease (approximately 3 beats per minute) in participants’ heart rate during the 6-minute time period immediately after conducting the breathing exercises (Spearman rank correlation coefficient –0.61, *P*<.001; S=9,816,176). There was no significant difference between the two groups for PHQ-9 and GAD-7 scores over the course of the semester. Exit interview responses indicated that participants perceived that the mobile Biofeedback app improved their health and helped them address stress challenges. All participants reported that the intervention helped them manage their stress better and expressed that health coaching via a mobile device would improve their overall health.

**Conclusions:**

Participants reported a positive perception of the app for their mental health self-management during a stressful semester. Future work should examine long-term effects of the app with a larger sample size balanced between male and female participants, randomized participant allocation, real-time detection of mental health symptoms, and additional features of the app.

## Introduction

Biofeedback is a technique to provide information of one’s bodily states that would otherwise be unknown to the person [1]. Biofeedback has been used to facilitate the self-regulation of involuntary physiological functions such as heart rate or heart rate variability (HRV) through real-time feedback [2]. In typical biofeedback training, trainees’ physiological functions are detected through sensors and displayed as visual or auditory information [3-5]. The trainees then learn how to self-regulate their physiological states through physical activity, breathing exercises, or meditation while monitoring their own biofeedback [6,7]. Biofeedback has long been used as a therapeutic measure to remediate a wide range of health issues [8]. Specifically, biofeedback has shown its effect in managing mental health symptoms such as stress, anxiety, and depression [9-11]. In addition, previous studies [12,13] have demonstrated the effectiveness of HRV biofeedback in reducing the severity of posttraumatic stress disorder (PTSD) among veterans. The efficacy of conventional biofeedback training to alleviate mental health symptoms has largely been examined through participants’ self-driven biofeedback practice, along with sporadic lab assessments of physiological conditions and perceived mental health symptoms [6,11,12]. While the effects of biofeedback methods were largely demonstrated during the lab sessions, evidence on its applicability to participants’ everyday situations has been limited [9].

Mobile health (mHealth) is the use of electronic technologies to deliver health care to patients and aims to enable the self-management of health conditions [14]. Health coaching is a process that utilizes education and feedback on a person’s health-related behaviors to help them self-manage their health [15]. Health coaching has traditionally been conducted via direct interaction between a patient and a health care professional [16]; however, digital health coaching using mHealth technologies is gaining popularity in its application to manage adverse health conditions [17].

The advent of mHealth technologies offers a variety of opportunities to address the limitation of the traditional biofeedback practice. Such capabilities include real-time monitoring of physiological measures, self-reporting of perceived mental health symptoms, and proactive intervention to manage health problems [18]. Recent studies have demonstrated the use of smartphone apps to facilitate stress recovery through biofeedback, including a smartphone app designed for breathing-based biofeedback to recover from a stressful task [19,20]. These studies found significantly lower levels of physiological stress markers among participants who used the smartphone app than among those who did not. Similarly, another study [21] used a smartphone app with meditation and self-reporting features aimed at capturing participants’ HRV and other physiological data. In addition, wearable technologies such as smartwatches further enable the same features of the smartphone apps to be provided in a more convenient and accessible manner [22]. However, existing mobile app–based biofeedback training usually involves controlled experiments conducted within laboratory settings. Therefore, there is an evident need to understand the effectiveness of mobile biofeedback apps for the self-management of users’ stress during their everyday lives.

Benefits of a mobile and wearable biofeedback technology are especially relevant for vulnerable populations such as college students and veterans. College students face reportedly rising mental health concerns, with evidence of a sharp rise in the severity and prevalence of mental disorders such as anxiety and depression, and can therefore benefit from mHealth solutions [23-26]. Students’ mental health has been a serious concern in higher educational institutions, and mental illnesses can affect students’ motivation, concentration, and social interactions, which are all crucial factors for students to succeed in higher education [27]. An analysis of a US national data set between 2005 and 2017 identified a drastic increase in depression, anxiety, and other mental health concerns among young adults aged 18 to 25 in recent years [28]. Additionally, severe mental health issues have been consistently observed among veterans, including an increased prevalence of PTSD and depression [29,30].

The purpose of this pilot study was to evaluate the feasibility of using a mobile biofeedback app to deliver health coaching on the proper deep-breathing technique for the self-management of stress in students’ everyday life situations. An mHealth coaching app called “Biofeedback” was developed to assist users in better managing their mental health through the practice of coached biofeedback training sessions.

It should be noted that the COVID-19 pandemic arose as an unexpected circumstance during the course of this study. Due to the physical distancing and other control measures placed on students and staff at the university and local communities, participants were not allowed to be brought into the lab after the study was initiated. Such pandemic-related restrictions offered a unique opportunity to test both the digital remote capabilities of the Biofeedback app outside of direct research lab contact and its ability to help users mitigate stress during an especially stressful time period.

## Methods

### Study Design

Participants were assigned to one of two groups based on the type of mobile phone they were using since the Biofeedback app was only compatible with iOS devices. Users of an iOS-based smartphone were assigned to an intervention group that received an Apple smartwatch paired with their smartphone with the Biofeedback app installed; users of other types of phones were assigned to a control group that did not receive the smartwatch (and thus no Biofeedback app). For the participants in the intervention group, counts of the coached breathing-exercise feature using the Biofeedback app were collected via the app. In addition, the heart rate of the participants in the intervention group was recorded by the app continuously while they wore the smartwatch. Heart rate was used as an indication of mental health status since stress can be measured as a function of physiological parameters such as heart rate and breathing rate [31,32]. Perceived mental health symptoms such as anxiety and depression were measured for both groups through self-reported responses to the 9-item Patient Health Questionnaire (PHQ-9) for depression [33] and the 7-item Generalized Anxiety Disorder (GAD-7) for anxiety [34,35], delivered through web-based surveys in approximately 2-week intervals for each group.

Table 1 outlines the type of measurement factors used in this study for each group of participants. At the end of the study, an exit interview and short survey were conducted to obtain the participants’ overall experience with the deep-breathing exercises, user feedback on the Biofeedback app, and recommendations for future design features of the app.

**Table 1:** Types of measurements between participant groups.

Measurement	Control group	Intervention group
Lab session	Once (1st session)	Once (1st session)
Web-based questionnaires (PHQ-9^a^ and GAD-7^b^)	4 times (2-week interval)	4 times (2-week interval)
Heart rate monitoring	Not monitored	Continuously measured while wearing smartwatch
Breathing exercise	As needed without Biofeedback app	As needed with Biofeedback app

^a^PHQ-9: 9-item Patient Health Questionnaire.

^b^GAD-7: 7-item Generalized Anxiety Disorder questionnaire.

### Participants

Participants were recruited from the student population at a large university in Texas. To be eligible for the study, participants had to be aged 18 years or older and enrolled as students with veteran status. Participants were recruited through dissemination of the study announcement across the veterans’ division of student affairs of the university.

### mHealth Coaching Intervention

**Figure 1:**
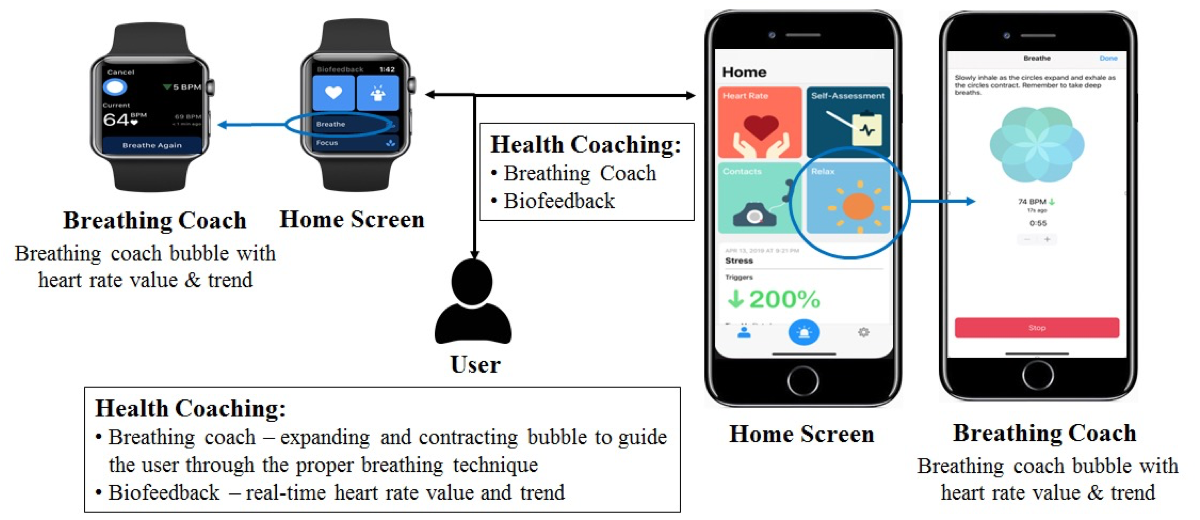
Overview of the Biofeedback app mobile health intervention.

The health coaching intervention for this study consisted of education on the proper deep-breathing technique in the lab followed by breathing exercises conducted during the participants’ daily lives. The Biofeedback app coached the participants through the proper deep-breathing technique while providing real-time feedback of their heart rate value and heart rate trend. Figure 1 provides an overview of the mHealth intervention for the intervention group. The participants in this group selected the breathing coach on the smartwatch or smartphone, and the coaching bubble then guided the participants through the proper deep-breathing process by slowly expanding to guide the participants through the inhalation process and slowly contracting to guide the participants through the exhalation process. Real-time heart rate was measured by the smartwatch and displayed on the smartwatch or smartphone screen along with a directional arrow to indicate an increasing trend (up arrow) or decreasing trend (down arrow) in heart rate.

### Study Procedure

All participants attended an initial lab session that lasted approximately 60 minutes. During the lab session, informed consent was obtained from the participants, demographics data were collected, and participants completed the PHQ-9 and GAD-7 surveys. Participants in both the intervention and control groups were given instructions on the biofeedback practice, including the proper deep-breathing method for managing stress. During this session, the HeartMath emWave Pro device [36] was used for HRV measurement and coherence [37]. The device detected and measured HRV noninvasively through a pulse sensor attached to the participant’s ear lobe. Coherence, as computed by HeartMath’s algorithm, is a measure of how wave-like the HRV patterns are. High coherence indicates a synchronization between sympathetic and parasympathetic systems, reflecting a stable status of the body to cope with stress.

The lab session consisted of three subsessions to measure coherence under different physiological conditions: (1) resting, (2) mental computation, and (3) deep breathing. In the first subsession, a baseline coherence score in a resting condition was measured for 2 minutes while participants remained seated, listening to nature sounds. In the second subsession, participants were given a mental computation task where they iteratively subtracted 7 from 2876 (ie, 2869 to 2862 to 2855, etc) for 2 minutes. The mental computation task was intended to create a stressful situation for the participants. In the third subsession, participants were instructed to practice a deep-breathing exercise (6 breaths per minute) for 2 minutes. The intervention group received additional training on the Biofeedback app to practice coached breathing while being provided real-time biofeedback data (eg, heart rate and heart rate trend).

After completing the first lab session, participants in the intervention group were asked to wear the smartwatch and use the features of the Biofeedback app in their daily lives. Heart rate data for the participants in the intervention group were continuously collected while the participants were wearing the smartwatch. The original design of the study included monthly lab sessions with all participants to measure coherence for the three scenarios previously discussed. However, due to the COVID-19 pandemic, these follow-up lab sessions were not permitted. At the end of the study, participants from both groups participated in a remote exit interview (via web-based video conferencing) to provide their experiences and opinions about the Biofeedback app and impacts of COVID-19 on their mental health. Table 2 lists the questions used for the exit interview.

**Table 2:** Questions used for the exit interview.

Question			Intervention group		Control group
**Biofeedback app usage**					
	How often and for how long did you perform the breathing exercises?	✓		✓	
	Did you use the breathing app on the smartwatch to guide you through the breathing exercises?	✓			
	Did you use the breathing app on the smartphone to guide you through the breathing exercises?	✓			
	Which device did you prefer (smartwatch or smartphone)? Please explain the reasons for your preference.	✓			
	Did you use any mobile apps or tools to guide you through the breathing exercises? If so, please describe the tool you used.			✓	
	What do you think are the potential benefits of the breathing app (as part of health coaching)?	✓		✓	
	What behaviors or health conditions do you think would benefit from health coaching?	✓		✓	
	What features would you want to have available to you for health coaching via a mobile device?	✓		✓	
	How much do you think health coaching via a mobile device would improve your overall health?	✓		✓	
**Impacts of COVID-19**					
	How did the COVID-19 situation affect your general stress levels during the semester?	✓		✓	
	Did the breathing exercise help in coping with stress during this period?	✓		✓	

### Outcome Variables

#### Coherence Scores During the First Lab Session

A repeated-measures ANOVA was performed to examine if there was any significant difference between the coherence scores for the three subsessions (ie, resting, mental computation, and deep breathing). A significance level of *P*<.05 was used for all statistical analyses. The Bonferroni correction method was used for posthoc analyses. All statistical analyses were conducted using R software version 3.6.1.

#### Average Heart Rate Before and After Breathing Exercises in Daily Living

To investigate the immediate effects of coached breathing exercises on stress in the participants’ daily lives, windows of the heart rate data immediately before and after the breathing exercises were extracted. We took a single 3-minute window of the heart rate data before a breathing exercise and 6 minutes of heart rate data after the exercises, divided into two windows of 3 minutes (0-3 minutes, 3-6 minutes). A trend analysis was performed using Spearman rank correlation to investigate the trends in heart rate data before and after the breathing exercises. Spearman rank correlation is a nonparametric test used to detect a monotonic trend in time-series data. A negative Spearman correlation coefficient corresponds to a decreasing trend and a positive coefficient indicates an increasing trend.

**Figure 2:**
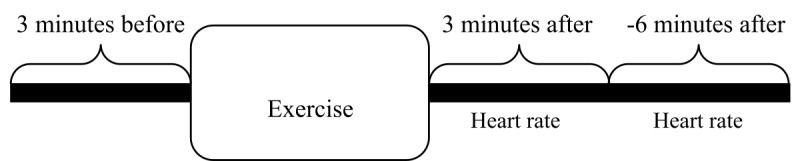
Heart rate data windowing process.

Figure 2 illustrates the windowing process of heart rate data extraction immediately before and after a breathing exercise. Average heart rates for each window were calculated. A repeated-measures ANOVA was performed to understand the effect of breathing exercises on the average heart rate. Following the repeated-measures ANOVA, Bonferroni posthoc tests were applied.

#### PHQ-9 and GAD-7 Scores

A mixed ANOVA was performed to identify significant difference in PHQ-9 and GAD-7 scores between the two groups over the semester.

#### Biofeedback App Engagement

Relationships between the questionnaire scores and Biofeedback app usage were further examined. Due to the small sample size, it was not feasible to perform a statistical analysis of these relationships. Thus, our analysis was simply focused on observing visual patterns in the relationships between Biofeedback app usage and the PHQ-9 and GAD-7 scores. Specifically, the individual participants’ usage patterns before and after the campus shutdown due to the COVID-19 pandemic were analyzed.

### Ethical Considerations

Research protocols for this quasi-experiment were approved by the Texas A&M University Institutional Review Board (IRB2018-0681D). In the initial lab session, individual participants were provided a written informed consent form. For survey and interview data, personally identifiable information such as each participant’s name was coded and deidentified (eg, “participant A”). Each participant received monetary compensation at three milestones: US $40 after completing the third online survey, US $60 after completing the fifth online survey, and US $100 after finishing the exit interview.

## Results

### Participants

**Figure 3:**
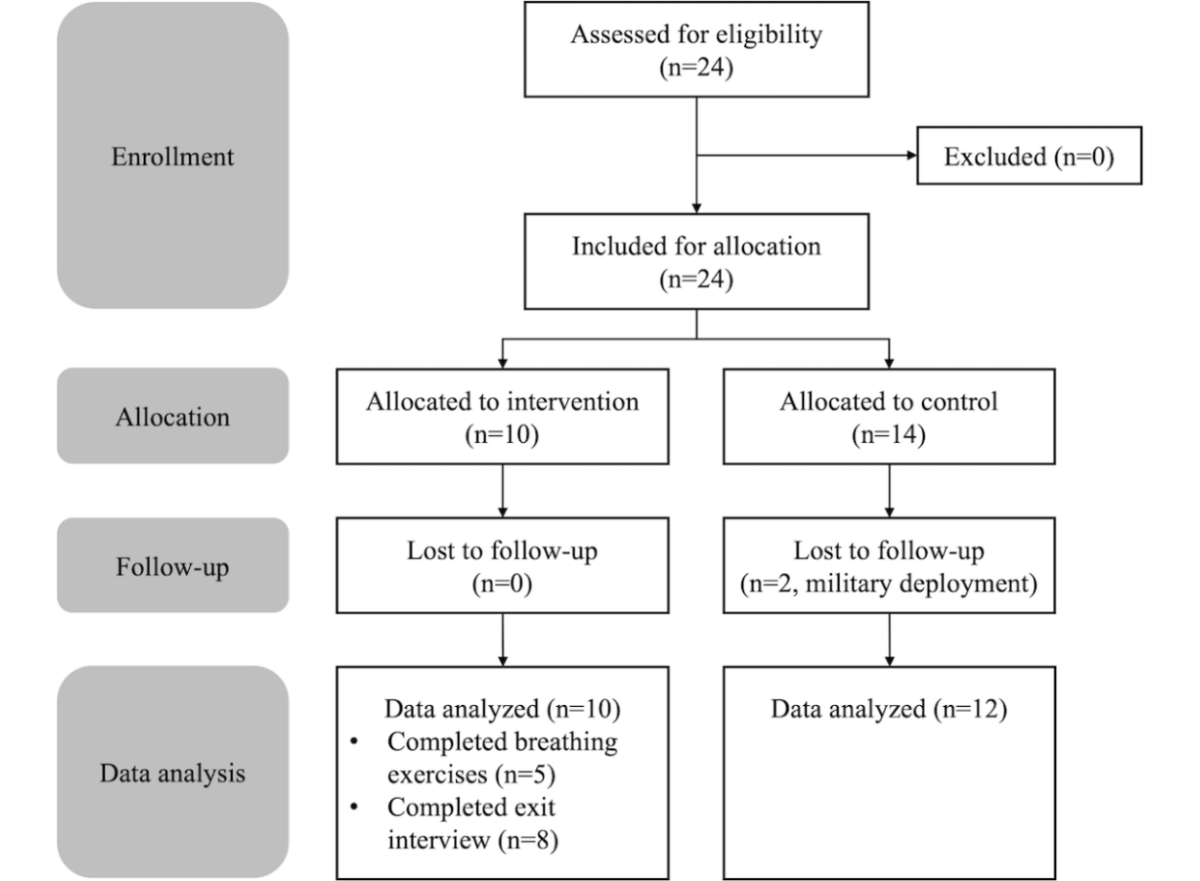
Flow diagram for participant enrollment, allocation, and analysis.

Twenty-four college student veterans were recruited for the study (22 males and 2 females) with a mean age of 28.2 years (SD 5.3, range 19-40). Ten participants were assigned to the intervention group (9 males and 1 female) and 14 were assigned to the control group (13 males and 1 female) depending on the type of smartphone they used. During the study, two participants in the control group withdrew due to military deployment orders. A total of 22 participants completed the study with 20 participants completing the exit interview (two participants in the intervention group chose not to participate in the exit interview). Out of 10 participants in the intervention group, 5 participants routinely performed the breathing exercises with heart rate feedback. The usage rate of the app remained fairly constant over the duration of the study for four of these participants, with usage increasing for one participant after the campus was closed due to the COVID-19 pandemic. Five participants did not complete any breathing exercise and were therefore excluded from the heart rate analysis. Table 3 shows the participant demographics and Figure 3 shows a diagram of the participant flow in the study adapted from the CONSORT (Consolidated Standards of Reporting Trials) standard [38].

**Table 3:** Participant demographics (N=24).

Characteristic		Value
Age (years), mean (SD)		28.2 (5.3)
**Gender, n (%)**		
	Male	22 (91.7)
	Female	2 (8.3)
**Academic year, n (%)**		
	Freshman	4 (16.7)
	Sophomore	6 (25)
	Junior	3 (12.5)
	Senior	7 (29.2)
	Graduate student	3 (12.5)
	No response	1 (4.2)

### Outcomes

#### Coherence Scores During the First Lab Session

Repeated-measures ANOVA showed a significant difference of the coherence scores among the three subsessions (resting, mental computation, and deep breathing) during the initial lab session (*F*_2,42_=9.188, *P*<.001, ƞ^2^=0.336). Bonferroni posthoc analysis showed significant differences between all pairs of three subsessions: resting and mental computation (*P*<.001), resting and breathing exercise (*P*=.003), and mental calculation and breathing exercise (*P*=.03).

#### Average Heart Rate Before and After Breathing Exercises in Daily Living

From the participants of the intervention group, 565 breathing exercises were recorded by the Biofeedback app. Thus, 1130 (565×2) windows of heart rate data were extracted for 3 minutes before and 6 minutes after the coached breathing exercises. Results from the Spearman rank correlation analysis showed a significant decreasing trend in the participants’ heart rate after performing the breathing exercise with an average Spearman rank correlation coefficient of –0.61 (*P*<.001 and S=9,816,176) for the 6-minute windows after the breathing exercise.

An initial analysis from the repeated-measures ANOVA indicated that participants’ heart rate in general did not have a clear change pattern around the breathing exercise (*F*_2,565_=0.306, *P*=.74). To take a deeper look into heart rate trends around the breathing exercises, we categorized the windows of heart rate before the breathing exercises into two groups: low heart rate (average heart rate in a window<80 beats per minute [bpm]) and high heart rate (average heart rate in a window≥80 bpm) in line with the thresholds used in the literature [39,40]. The results from repeated-measures ANOVA showed that when the average heart rate before the breathing exercise was over 80 bpm, the heart rate dropped significantly after breathing exercises (*F*_2,56_=11.032, *P*<.01, *d*=0.079). The Bonferroni posthoc test was then performed to look into significant differences between windows (3 minutes before the exercise, 3 minutes following the exercise, and 3-6 minutes after the exercise). The results from the posthoc test (Table 4) showed that when the average heart rate before the breathing exercises was over 80 bpm, it dropped 3 bpm after the exercise on average. There was no significant difference between 3 minutes following the exercise and 3-6 minutes after the breathing exercise.

**Table 4:** Bonferroni posthoc test results.

Window 1	Window 2	Mean difference (Window 1–Window 2)	*df*	*P* value
3 minutes after breathing exercise	3-6 minutes after breathing exercise	1.67	56	.10
3 minutes after breathing exercise	3 minutes before breathing exercise	–3.09	56	.003
3-6 minutes after breathing exercise	3 minutes before breathing exercise	–3.76	56	<.001

#### PHQ-9 and GAD-7 Scores

**Figure 4:**
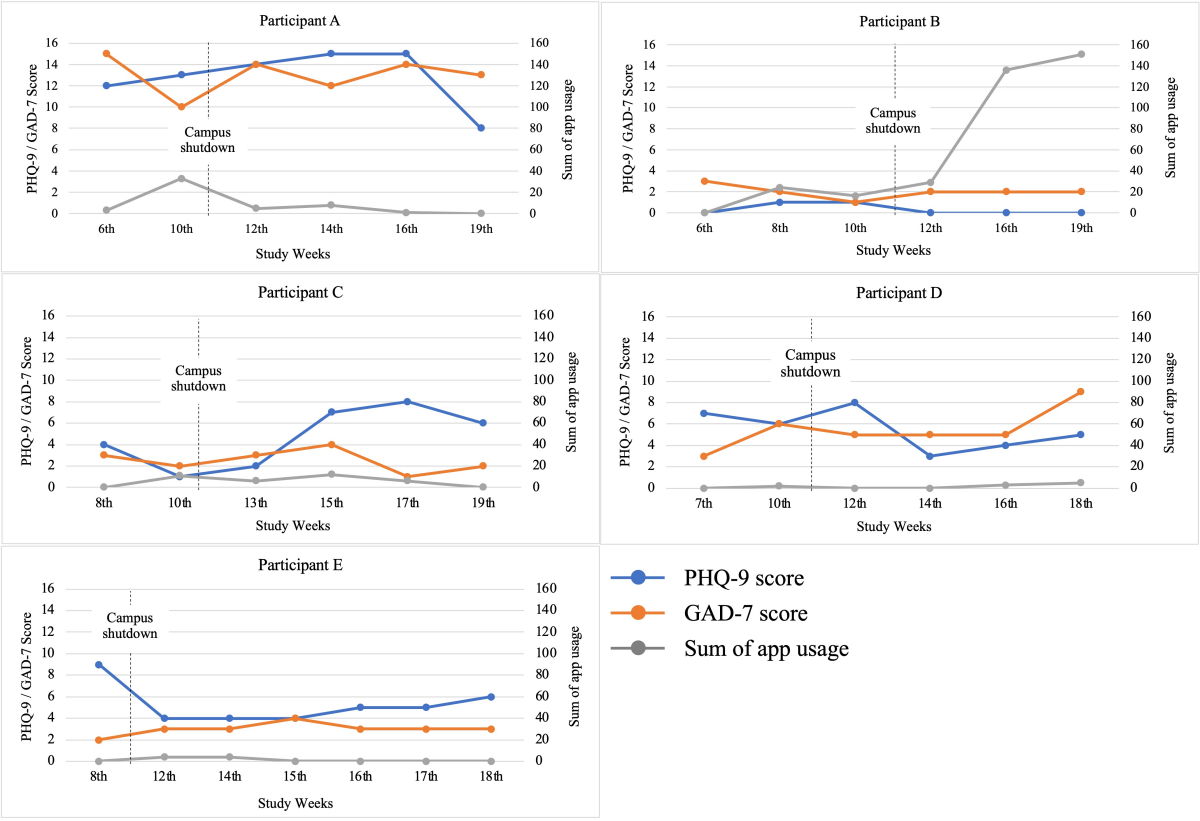
Patient Health Questionnaire-9 (PHQ-9) and General Anxiety Disorder-7 (GAD-7) scores and Biofeedback app engagement for intervention group participants.

There was no significant difference between the intervention and control groups for the PHQ-9 and GAD-7 mean scores during the study. In addition, there was no significant change of the PHQ-9 and GAD-7 mean scores within each group over the semester period. However, we found that there were different types of the Biofeedback app users in relation to the PHQ-9 and GAD-7 scores. Figure 4 presents the associations of the frequency of Biofeedback app usage and PHQ-9 and GAD-7 scores during the semester for the five participants of the intervention group who completed the breathing exercise using the Biofeedback app at least once. Participant A reported the highest mental health symptoms (PHQ-9 score>9, mild to moderate depression; GAD-7 score>10, moderate anxiety) during the semester. However, these data should also be interpreted in relation to the Biofeedback app usage and exit interview comments of each participant (see the following subsection).

#### Biofeedback App Engagement

Out of 10 participants in the intervention group, 5 participants did not complete any breathing exercise. Therefore, these 5 participants were excluded from the Biofeedback app engagement analysis. Figure 4 illustrates the frequency of Biofeedback app usage for the 5 participants who completed coached breathing exercises (Participants A, B, C, D, and E) compared with their corresponding PHQ-9 and GAD-7 scores over the course of the study.

Participant A reported the highest mental health symptoms (PHQ-9>9, mild to moderate depression; GAD-7>10, moderate anxiety) during the semester. However, their Biofeedback app usage dropped after the campus was shut down in the 11th week of 2020 due to the COVID-19 pandemic. During the exit interview, Participant A stated:

Yes, it [COVID-19] did [impact general stress levels] a lot: uncertainty what was happening and not just in my life but school and work. Note I used breathing exercise app less the more I got stressed with the situation. Uncertainty of schedule changed when I was out of school. I didn’t have my regular routine.

Participant B showed a huge increase in Biofeedback app usage after the campus closure although the participant reported low PHQ-9 and GAD-7 scores (<5 points, minimal depression and anxiety) throughout the semester. During the exit interview, Participant B stated:

Not much change to the social dynamic because you don’t get much time to do whatever you want during the semester anyway. Transition to online classes was a bit stressful. Being stuck at home, knowing that I had all the time to myself, helped me plan, including the time for the breathing exercise.

Participant C exhibited largely an opposite pattern to that of Participant B, presenting increased PHQ-9 scores (<9 points, mild depression) after the campus shutdown and low app usage throughout the semester (fewer than 20 times per week). During the exit interview, Participant C stated:

It was wave function like, up a couple of weeks, down a few weeks. Injury affected my stress as well. [It] wasn’t like a normal semester.

Participant D did not show a particular pattern either in the app usage or reported mental health symptoms. During the exit interview, Participant D stated:

I remained resilient to the effect of COVID-19. [It] didn’t cause disturbance to daily life. Online was easy to work with, low impact. More stressful events around 6th week of being restricted to your household.

Participant E did not show a particular pattern either in the app usage or reported mental health symptoms. During the exit interview, participant E stated:

[COVID-19] did not affect the stress during the semester. Personal experience in the past years.

#### Additional Exit Interview and Survey Data

Exit interviews were conducted with 20 of the participants, including 8 in the intervention group and 12 in the control group, using the questions in Table 2. Participants in the intervention group provided feedback based on their user experience of the Biofeedback app. Participants in the control group were provided a demonstration of the functions and features of the Biofeedback app as part of the exit interview to solicit input on the Biofeedback app. Most participants (18/20) reported an overall positive perception of health coaching via a mobile app.

All participants (20/20) reported that this study helped them manage their stress better. All participants opined that health coaching via a mobile device would improve their overall health a great deal (4/20), a lot (5/20), a moderate amount (9/20), and a little (2/20), with none reporting that it would have no help at all. Both groups reported similar perceived benefits of health coaching via a mobile app: providing health-related reminders (8/20), promoting self-accountability for managing one’s health (7/20), managing health conditions (5/20), managing stress (4/20), providing health coaching instructions (4/20), and improving health-related behaviors (2/20). Participants identified the following additional desired features for a health coaching mobile app: reminders (14/20), historical data/trends (8/20), health-related educational material (4/20), instructions on how to perform various calming exercises (4/20), capability of sensing stress and prompting the user to conduct breathing exercises (3/20), activity tracker (3/20), and additional calming exercises (3/20). Participants in the intervention group preferred the smartwatch app over the smartphone app (5/8) due to ease of use.

Lastly, we found varied impacts of COVID-19 on the participants’ stress level. While 13 out of 20 participants (5 from the intervention group and 8 from the control group) reported a slight to significant increase in their stress level during the COVID-19 pandemic, 6 participants (3 from the intervention group and 3 from the control group) answered that their stress actually decreased during the semester. Despite the varied impacts of the COVID-19 pandemic on the participants’ daily lives, a majority (17/20) indicated that the breathing exercises helped in coping with stress during the pandemic.

## Discussion

### Principal Findings

This study evaluated the usage of the mobile Biofeedback app as a semester-long intervention for mental health challenges among college student veterans. Results of the initial lab session indicated that deep-breathing exercises alleviated participants’ stress (ie, increased coherence score) caused by the mental computation task. This finding confirms the effect of breathing exercises to reduce mental health symptoms in line with previous studies conducted in a controlled environment [6,7].

Furthermore, our study identified the benefits of the mobile Biofeedback app in participants’ actual day-to-day lives. Results of the intervention group participants’ daily use of the app revealed that the breathing exercise significantly decreased their heart rate immediately after the exercise (0 to 3 minutes and 3 to 6 minutes after the exercise compared with 3 minutes before the exercise). These findings support that biofeedback techniques such as deep-breathing exercises can be useful to manage stressful situations in participants’ daily lives beyond the lab setting. In particular, our study showed promise for health coaching via the mobile Biofeedback app as provided via smart devices (eg, smartwatches and smartphones).

Our study did not detect any significant difference of PHQ-9 and GAD-7 scores between the intervention and control groups and identified only short-term effects (up to 6 minutes) after a breathing exercise on reducing the participants’ heart rate. Therefore, additional studies are required to examine whether the coached breathing exercises guided by the mobile Biofeedback app actually bring positive impacts on perceived mental health symptoms (PHQ-9 and GAD-7) and long-term mental health improvement in participants’ daily lives. Additionally, effects of complementary biofeedback techniques such as mindfulness training and meditation [41] need to be investigated in the context of users’ daily use of mHealth coaching apps over a long-term period. Our findings suggest that more efforts are needed to develop mobile mental health apps to support vulnerable groups (eg, college students, veterans) in dealing with mental health–related challenges.

### Limitations and Insights for Future Work

This study has several limitations. First, breathing exercises were not consistently practiced over the study period. The COVID-19 pandemic had unexpectedly impacted the participants’ breathing practices by preventing participants from having the planned follow-up monthly lab sessions with the research team. The format of our study, which was based on the participants’ self-managed breathing exercises in a natural environment, did not afford the research team’s control over participants’ consistent practice of the breathing exercise. For more conclusive evidence, future studies need to be conducted over a stable period where no major societal disruptions (eg, pandemic events) occur.

Second, our study did not allow for comparison of physiological measures (eg, heart rate) between the intervention and control groups because the mobile Biofeedback app was only provided to the participants who were using an iOS-based smartphone. Providing the smartwatch devices to all participants would allow for recording of heart rate, and thereby comparison between the two groups.

Third, physiological measures were obtained by different means between the lab and natural settings. While the biofeedback practice in the initial lab session was based on HRV, the Biofeedback app used by the intervention group was limited to heart rate data due to the current capability of the smartwatch. Hence, future research should use consistent measures across lab-based experiments and self-administered exercises under natural living conditions. However, it is worth noting that existing HRV measurement devices require a separate sensor such as an ear lobe sensor or a chest strap to be worn by the participant. Requiring the participant to wear multiple sensors regularly and continuously in their daily lives may not be feasible with the current smartwatch devices. To address this issue, an HRV measurement feature is recommended for future smartwatch devices.

Fourth, the number of participants (n=10 in the intervention group and n=12 in the control group) was not sufficient to detect significant differences between study conditions. It should be noted that two participants from the control group were not able to finish the participation due to military deployment. There was also a large imbalance between male (n=22) and female (n=2) participants. Issues with the recruitment of study participants appear to be largely due to the inherent characteristics of the target population, student veterans. According to the 2020 National Center for Veterans Analysis and Statistics [42], 89.6% of all veterans were male and 10.4% were female. In addition, the Institute for Veterans and Military Families indicated that 3%-4% of US college students are veterans [43]. To address these limitations, future studies need to consider recruiting participants from broader populations such as veterans in local communities and student veterans from multiple institutions.

Lastly, some of the participants in the intervention group had no or low completion of the breathing-exercise feature of the Biofeedback app. A systematic literature review of user engagement in mental health apps [44] identified the low sustained use of mobile mental health apps and lack of usability testing standards as persistent challenges. To address these challenges, future studies are required to examine ways to evaluate and increase users’ engagement with the mobile Biofeedback app and their execution of biofeedback techniques (eg, deep breathing).

Despite the limitations mentioned above, it should be acknowledged that this study was a feasibility study where the research team developed a novel biofeedback mobile app and conducted a pilot test to student veterans, a subset of a large veteran population. Furthermore, the COVID-19 pandemic created unexpected and significant disruptions to interactions with the participants as well as the participants’ daily lives. Based on the formative findings and limitations revealed in this study, future research is warranted to further evaluate the efficacy of using mobile mental health platforms to deliver biofeedback interventions and address the growing mental health needs of various vulnerable populations.

### Conclusions

This study examined student veterans’ usage of the mobile Biofeedback app and identified short-term effects of the breathing exercises to manage stress during COVID-19. Although additional research efforts are required to further confirm the long-term benefits of the mobile Biofeedback app, our study has suggested that coaching exercises on the proper breathing technique result in a significant decrease in average heart rate immediately following the breathing exercise. In addition, participants had a positive perception of the mobile mental health coaching system to manage their everyday challenges. With additional efforts for the timely detection of mental health symptoms and personalized biofeedback interventions, mobile biofeedback technology is expected to serve as an effective solution to growing mental health issues among vulnerable populations and in future pandemic events.

## Abbreviations

bpm: beats per minute
CONSORT: Consolidated Standards of Reporting Trials
GAD-7: 7-item Generalized Anxiety Disorder questionnaire
HRV: heart rate variability
mHealth: mobile health
PHQ-9: 9-item Patient Health Questionnaire
PTSD: posttraumatic stress disorder

## Data Availability

The data sets generated during and/or analyzed during the current study are not publicly available because the data are concerned with mental health issues of the participants, but are available from the corresponding author on reasonable request.
